# Oral Health–Related Quality of Life and Patient-Reported Outcomes After Implant Rehabilitation Using CAS Kit–Assisted Indirect Maxillary Sinus Augmentation: A Longitudinal Observational Study

**DOI:** 10.12688/f1000research.179345.2

**Published:** 2026-05-25

**Authors:** Sanath Kumar Shetty, Mallikarjuna Ragher, Rajesh Shetty, Savitha Dandekeri, Sunaina M, Nafiya Abdul Aziz

**Affiliations:** 1 PROSTHODONTICS, Yenepoya (Deemed to be University) Dental College, Mangaluru, Karnataka, India; 2 PROSTHODONTICS, Yenepoya (Deemed to be University) Dental College, Mangalore, Karnataka, 575018, India; 3 PROSTHODONTICS, Yenepoya (Deemed to be University) Dental College, Mangalore, Karnataka, 575018, India; 4 PROSTHODONTICS, Yenepoya (Deemed to be University) Dental College, Mangalore, Karnataka, 575018, India; 5 PROSTHODONTICS, Yenepoya (Deemed to be University) Dental College, Mangalore, Karnataka, 575018, India; 6 PROSTHODONTICS, Yenepoya (Deemed to be University) Dental College, Mangalore, Karnataka, 575018, India

**Keywords:** Maxillary Sinus Augmentation; Dental Implants; Quality of Life; Patient-Reported Outcome Measures; Posterior Maxilla; Minimally Invasive Surgical Procedures

## Abstract

**Background:**

Partial edentulism in the posterior maxilla is frequently complicated by alveolar bone resorption and maxillary sinus pneumatization, limiting implant placement and negatively affecting oral health–related quality of life (OHRQoL). Minimally invasive sinus augmentation techniques, such as CAS KIT–assisted indirect sinus elevation, aim to reduce surgical morbidity; however, evidence regarding patient-reported outcomes and quality of life following such interventions remains limited.

**Objective:**

To evaluate oral health–related quality of life and patient-reported outcomes following implant rehabilitation using CAS KIT–assisted indirect maxillary sinus augmentation.

**Materials and Methods:**

This longitudinal observational study included 34 patients who underwent CAS KIT–assisted transcrestal sinus augmentation with implant placement in the posterior maxilla. Postoperative recovery and satisfaction were assessed using the HRQOLquestionnaire over seven postoperative days. OHRQoL was evaluated using the OHIP-14 questionnaire at baseline and one month after prosthetic rehabilitation. Data were analyzed using repeated measures ANOVA with a significance level set at p < 0.05.

**Results:**

HRQOLscores showed a statistically significant improvement over the seven-day postoperative period (p < 0.001), with stabilization observed from Day 5 onward. OHIP-14 scores demonstrated a highly significant reduction from baseline to post-intervention assessment (p < 0.001), indicating marked improvement in OHRQoL across all participants.

**Conclusion:**

CAS KIT–assisted indirect sinus augmentation followed by implant rehabilitation results in rapid postoperative recovery and significant improvement in patient-reported outcomes, supporting its role as a predictable and patient-centered treatment modality for posterior maxillary rehabilitation.

## Introduction

Partial edentulism in the posterior maxilla remains a prevalent clinical condition with significant implications for oral health–related quality of life (OHRQoL). ^1^ Individuals with fewer than 20 natural teeth consistently report poorer OHRQoL compared with those retaining functional dentitions, as tooth loss compromises masticatory efficiency, speech, facial esthetics, and self-esteem, thereby influencing nutritional intake, social interaction, and overall wellbeing. ^2^ Following tooth extraction, the posterior maxilla undergoes progressive alveolar bone resorption accompanied by maxillary sinus pneumatization, frequently resulting in reduced vertical bone height that limits conventional implant placement. Epidemiological studies indicate that posterior maxillary bone deficiency is among the most common anatomical constraints encountered in implant dentistry, necessitating adjunctive bone augmentation procedures that may prolong functional disability and restrict treatment options. ^3– 5^

The evolution of maxillary sinus augmentation techniques reflects a progressive shift from invasive surgical procedures toward more conservative and predictable approaches. Traditionally, sinus floor elevation has been performed using either the lateral (direct) window technique or the crestal (indirect) approach. The lateral window technique permits substantial vertical bone augmentation and is indicated in cases of severe alveolar bone deficiency; however, it is associated with increased surgical trauma, longer operative time, higher postoperative morbidity, and delayed recovery. Conversely, the transcrestal approach was introduced as a less invasive alternative, offering reduced patient discomfort and faster healing, though earlier techniques were limited by restricted control over sinus membrane elevation. Advances in surgical philosophy have driven a paradigm shift toward minimally invasive sinus augmentation, emphasizing reduced tissue trauma, shorter procedure duration, lower complication rates, and enhanced patient comfort. This shift has been facilitated by innovations such as hydraulic pressure systems, balloon-assisted membrane elevation, and piezoelectric instruments, which improve the safety and predictability of Schneiderian membrane elevation. Among these developments, the Crestal Approach Sinus (CAS) Kit represents a significant advancement by enabling controlled, atraumatic, and minimally invasive transcrestal sinus elevation. Its calibrated instrumentation allows precise membrane elevation while minimizing the risk of perforation, aligning with contemporary trends in patient-centered implant therapy.

While clinical and radiographic outcomes of sinus augmentation—including implant survival rates, bone gain, and complication rates—are well documented, these parameters fail to capture patients’ subjective experiences. ^6– 8^ Contemporary dental research increasingly emphasizes patient-reported outcome measures (PROs) as essential indicators of treatment success, with OHRQoL representing a multidimensional construct encompassing functional limitation, physical pain, psychological discomfort, and social disability. Previous systematic reviews demonstrate that implant-supported prostheses improve oral health–related quality of life (OHRQoL) in edentulous and partially edentulous patients; however, evidence comparing different surgical modalities—particularly CAS-KIT assisted technique remains limited. It is unclear whether the improved surgical control associated with CAS-guided indirect sinus augmentation translate into a favorable patient-centered outcomes, including chewing efficiency, dietary habits, psychosocial wellbeing, and overall satisfaction.

Therefore, a significant knowledge gap exists regarding the impact of CAS KIT–assisted indirect maxillary sinus augmentation followed by implant rehabilitation on OHRQoL and PROs. The objective of this longitudinal observational study was to evaluate oral health–related quality of life and patient-reported outcomes in individuals who underwent implant rehabilitation following CAS KIT–assisted indirect maxillary sinus augmentation. The null hypothesis was that CAS KIT–assisted indirect sinus augmentation followed by implant rehabilitation would not result in significant improvements in OHRQoL, while the alternative hypothesis proposed that this minimally invasive approach would significantly enhance OHRQoL, yield high patient satisfaction, and exert minimal negative impact on daily functioning.

## Materials and methods

### Study design and ethical considerations

This longitudinal observational study was conducted to evaluate oral health–related quality of life (OHRQoL) and patient-reported outcomes following implant rehabilitation using CAS KIT–assisted indirect maxillary sinus augmentation. The cross-sectional design was chosen to assess patient-centered outcomes after completion of surgical and prosthetic treatment without modifying standard clinical care. Ethical approval was obtained from the Yenepoya Ethics Committee-2(Ref. YEC2/2024/342), Yenepoya (Deemed to be University) prior to study initiation, and the study was conducted in accordance with the Declaration of Helsinki (2013 revision). Written informed consent was obtained from all participants after providing detailed information in their native language. The study adhered to the STROBE guidelines for reporting longitudinal observational studies.

### Study population and setting

The study was carried out at the Centre for Oral Implantology a university-based tertiary care center, between June 2024 and September 2025. Thirty-four patients aged 18–60 years ( Table 1) requiring implant-supported rehabilitation in the deficient posterior maxilla with CAS KIT–assisted indirect sinus augmentation were recruited using simple random sampling. All participants completed implant placement followed by prosthetic rehabilitation and consented to participate in a questionnaire-based evaluation.

**Table 1.** Demographic characteristics of participants.

Characteristic	N (%) or Mean ± SD
Number of patients	34
Age (years)	42.3 ± 11.5
Sex	
– Male	18 (52.9%)
– Female	16 (47.1%)
Implant site	
– Right posterior maxilla	20 (58.8%)
– Left posterior maxilla	14 (41.2%)

### Sample size calculation

Sample size estimation was performed using G*Power software based on data from a previous study by Hadar et al. Power analysis for an independent t-test was conducted with a conventional effect size of 0.5, a type I error rate (α) of 5%, and a type II error rate (β) of 20%, corresponding to a power of 80%. The calculated minimum sample size required for the study was 34 participants.

### Surgical protocol

All participants underwent preoperative clinical evaluation and cone-beam computed tomography (CBCT) imaging to assess residual alveolar bone height and maxillary sinus anatomy. Digital planning was performed to guide implant positioning and depth control using the CAS KIT system. Indirect maxillary sinus augmentation was carried out via a transcrestal approach following the CAS KIT drilling protocol, with controlled elevation of the Schneiderian membrane and implant placement performed as per standard clinical indications. Postoperative care included routine medications, oral hygiene instructions, and scheduled follow-up visits.

### Prosthetic rehabilitation

Prosthetic rehabilitation was completed following an adequate healing period and confirmation of implant stability. Implant-supported restorations were fabricated and delivered based on clinical requirements, with the type of prosthesis and loading protocol determined according to patient-specific factors and standard implant prosthetic principles.

### Patient-reported outcome measures

Patient-reported outcomes were assessed using two validated questionnaires. Postoperative recovery and satisfaction were evaluated using the HRQOL questionnaire consisting of 19 items assessing oral function, general activity, other symptoms, and pain, recorded daily for seven postoperative days using a five-point Likert scale through structured telephonic interviews. Oral health–related quality of life was assessed using the OHIP-14 questionnaire at baseline - preoperatively before surgical procedure and at one month following prosthetic rehabilitation, with responses recorded on a five-point frequency scale and analyzed both as overall scores and across seven standardized subdomains.

### Data collection and quality assurance

All questionnaires were administered in the participants’ native language using validated translations. Measures were taken to ensure completeness and consistency of responses, and participants with incomplete or inconsistent data were excluded according to predefined withdrawal criteria. Collected data were anonymized, securely stored, and prepared for statistical analysis.

### Statistical analysis

Statistical analysis was performed using Statistical Package for the Social Sciences (SPSS) software, version 26.0 (IBM Corp., Armonk, NY, USA). Descriptive statistics, including mean, standard deviation (SD), standard error (SE), and coefficient of variation, were calculated for HRQOL and OHIP scores at different time points. Changes in HRQOL scores over the seven-day observation period were analyzed using repeated measures analysis of variance (ANOVA) to assess the effect of time. Following a significant ANOVA result, post hoc pairwise comparisons were performed to identify specific differences between individual days. Changes in OHIP scores between pre- and post-intervention assessments were also evaluated using repeated measures ANOVA. A p value <0.05 was considered statistically significant for all analyses.

## Results

**Graph 1.**
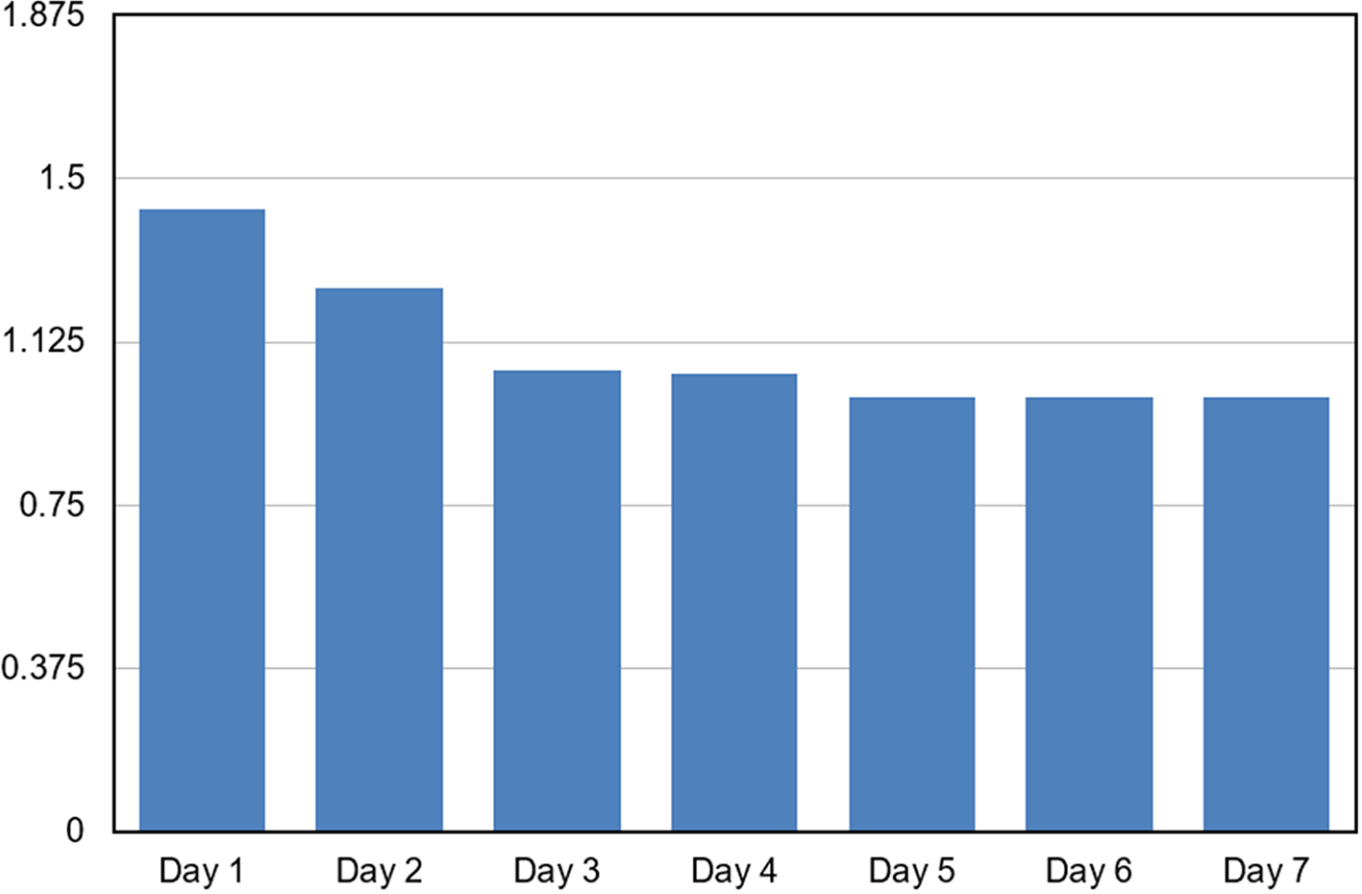
Graph depicting trend of Mean HRQOL Scores Over the 7-Day Observation Period.

The HRQOL scores demonstrated a significant improvement over the seven-day observation period. Repeated measures ANOVA revealed a statistically highly significant effect of time on HRQOL scores (F = 261.1, p < 0.001). The mean HRQOL score decreased progressively from Day 1 (1.429 ± 0.109) to Day 4 (1.053 ± 0.090), indicating an improvement in health-related quality of life over time. Post hoc pairwise comparisons showed significant differences between Day 1 and all subsequent days, as well as between Day 2 and Days 3–7 (p < 0.001). No significant difference was observed between Day 3 and Day 4 (p = 0.325). From Day 5 onwards, HRQOL scores stabilized at 1.000 with no inter-individual variability, and no significant differences were observed among Days 5, 6, and 7, indicating sustained improvement [ Tables 2- 4; Graph 1]. The stabilization of HRQOL scores observed during the later postoperative period reflects minimal postoperative discomfort and functional limitation reported by the majority of participants following healing and implant rehabilitation.

**Table 2.** Descriptive statistics of HRQOL scores across the 7-day follow-up period.

*Descriptive statistics*				
Day	Mean	SD	SE	Coefficient of variation
1	1.429	0.109	0.019	0.076
2	1.247	0.071	0.012	0.057
3	1.059	0.092	0.016	0.087
4	1.053	0.090	0.015	0.085
5	1.000	0.000	0.000	0.000
6	1.000	0.000	0.000	0.000
7	1.000	0.000	0.000	0.000

**Table 3.** Repeated measures ANOVA showing changes in HRQOL scores over time.

*Repeated measure ANOVA*					
Cases	Sum of squares	df	Mean square	F	p
HRQOL	5.540	6	0.923	261.1	*< .001*

**Table 4.** Post hoc pairwise comparisons of HRQOL scores between different time points.

*Post hoc comparisons – HRQOL*						
Day vs day		Mean difference	SE	df	t	p value
1	2	0.182	0.017	33	10.642	*< .001*
1	3	0.371	0.020	33	18.095	*< .001*
1	4	0.376	0.022	33	16.834	*< .001*
1	5	0.429	0.019	33	23.015	*< .001*
1	6	0.429	0.019	33	23.015	*< .001*
1	7	0.429	0.019	33	23.015	*< .001*
2	3	0.188	0.008	33	22.978	*< .001*
2	4	0.194	0.010	33	18.863	*< .001*
2	5	0.247	0.012	33	20.391	*< .001*
2	6	0.247	0.012	33	20.391	*< .001*
2	7	0.247	0.012	33	20.391	*< .001*
3	4	0.006	0.006	33	1.000	0.325
3	5	0.059	0.016	33	3.708	*0.005*
3	6	0.059	0.016	33	3.708	*0.005*
3	7	0.059	0.016	33	3.708	*0.005*
4	5	0.053	0.015	33	3.447	*0.006*
4	6	0.053	0.015	33	3.447	*0.006*
4	7	0.053	0.015	33	3.447	*0.006*
5	6	0.000	0.000	33	NaN	NaN
5	7	0.000	0.000	33	NaN	NaN
6	7	0.000	0.000	33	NaN	NaN

**Graph 2.**
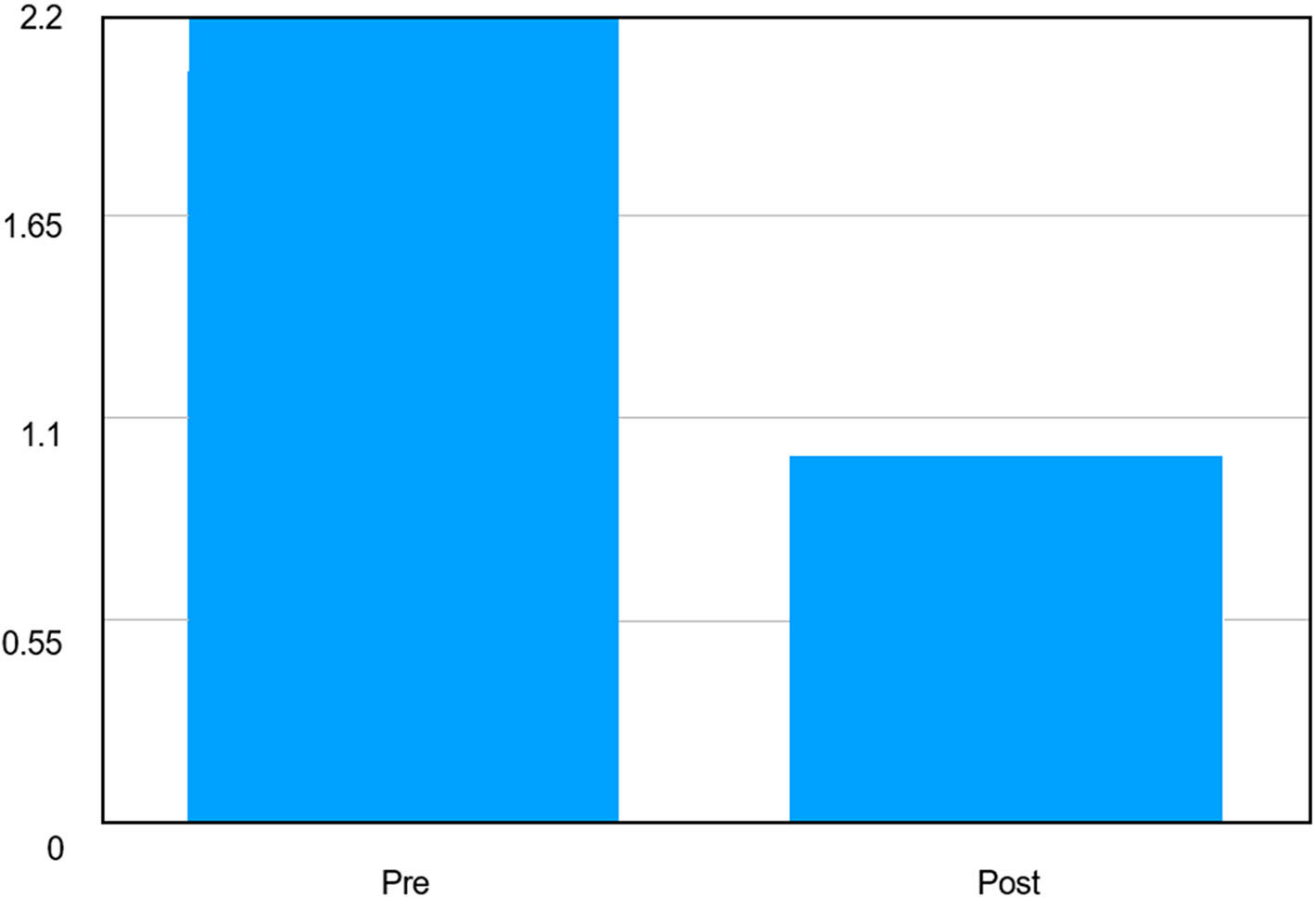
Graph depicting comparison of Mean OHIP Scores Before and After Intervention.

A statistically highly significant reduction in OHIP scores was observed from pre- to post-intervention assessment. Repeated measures ANOVA demonstrated a significant effect of time on OHIP scores (F = 1706, p < 0.001). The mean OHIP score decreased from 2.050 ± 0.148 at baseline to 1.000 ± 0.000 following the intervention, indicating a marked improvement in oral health–related quality of life. The absence of variability in post-intervention scores reflects uniform improvement across all participants [ Tables 5, 6; Graph 2].

**Table 5.** Descriptive statistics of OHIP scores at pre- and post-intervention assessments.

*Descriptive statistics*				
OHIP	Mean	SD	SE	Coefficient of variation
Pre	2.050	0.148	0.025	0.072
Post	1.000	0.000	0.000	0.000

**Table 6.** Repeated measures ANOVA comparing pre- and post-intervention OHIP scores.

*Repeated measures ANOVA*					
Cases	Sum of squares	df	Mean square	F	p
OHIP	18.743	1	18.743	1,706	*< .001*

## Discussion

This study demonstrated a statistically significant improvement in oral health–related quality of life following the intervention, thereby rejecting the null hypothesis and fulfilling the primary research objectives. Both HRQOL and OHIP-14 scores showed substantial reductions over the observation period, reflecting marked improvement in patient-reported outcomes. Although the CAS KIT technique is well established for transcrestal sinus elevation, limited literature is available regarding patient-reported postoperative quality-of-life outcomes following this minimally invasive approach. This study evaluates patient-centered outcomes following CAS KIT–assisted indirect sinus augmentation, offering novel evidence that this minimally invasive approach translates directly into measurable patient-reported benefits. The most pronounced improvement occurred during the early follow-up phase, with HRQOL scores demonstrating rapid and sustained normalization from Day 5 onwards, indicating high levels of patient satisfaction and consistent clinical success across the study population. The uniform post-intervention OHIP-14 scores further underscore the effectiveness of the intervention in minimizing the perceived impact of oral health on daily functioning. Collectively, these findings highlight favorable clinical outcomes, the presence of strong time-dependent effects as key predictors of improvement, and the significant value of incorporating patient-reported outcome measures into evaluations of minimally invasive sinus augmentation techniques.

The postoperative recovery pattern observed in the present study demonstrates a rapid improvement during the early healing phase (Days 1–4), followed by stabilization between Days 5 and 7, which is consistent with patient-reported outcome data from sinus augmentation literature. Rengo et al. reported moderate pain limited to the first two postoperative days with a progressive decline thereafter, alongside low discomfort scores and high willingness to repeat the procedure, indicating favorable short-term morbidity. ^9^ Similarly, Schiegnitz et al. showed that procedure-related complaints peak immediately postoperatively and significantly diminish over time. Compared with lateral window approaches, which are commonly associated with prolonged swelling, discomfort, and delayed recovery, transcrestal techniques have been shown to result in reduced morbidity and faster functional recovery. ^10^ These findings may be attributed to reduced surgical trauma, preservation of Schneiderian membrane integrity, and minimal bone manipulation inherent to indirect sinus elevation techniques. The accelerated recovery timeline observed in the present study appears comparable or favorable to conventional crestal approaches reported in the literature, supporting the clinical advantage of minimally invasive sinus augmentation strategies.

Beyond early recovery, the complete restoration of oral health–related quality of life observed post-intervention, reflected by a uniform OHRQoL score of 1.000, aligns with previous studies demonstrating significant improvements following implant rehabilitation in the posterior maxilla. The minimal variability observed during later postoperative intervals may indicate consistent patient adaptation and reduction in postoperative discomfort following healing. Schiegnitz et al. reported marked enhancement across functional, physical, and psychological OHIP domains following sinus augmentation, particularly in edentulous patients. ^10^ The multidimensional improvement observed—including functional limitation, psychosocial disability, and patient satisfaction—can be attributed to restored masticatory efficiency, improved esthetics, and enhanced psychosocial wellbeing, reinforcing the patient-centered benefits of successful sinus augmentation and implant-supported rehabilitation as outlined in the introduction.

At present, there is a clear absence of literature specifically evaluating patient reported outcomes following CAS Kit–guided sinus augmentation; however, meaningful inferences can be drawn by comparing its principles with established transcrestal techniques described in the literature. Comprehensive reviews, including that by Lafzi et al., consistently demonstrate that transcrestal approaches—particularly those employing drill-based, hydraulic, balloon-assisted, and piezoelectric modalities—are associated with lower postoperative morbidity, reduced membrane perforation rates, and faster patient recovery when compared with the lateral window technique. These advantages underpin the broader paradigm shift toward minimally invasive sinus augmentation. The CAS approach conceptually aligns with this evolution by emphasizing controlled sinus membrane elevation, elimination of malleting forces, and gradual, predictable manipulation of the sinus floor. Such controlled elevation mechanisms parallel those reported in hydraulic and balloon-assisted techniques, which distribute pressure evenly across the Schneiderian membrane and reduce focal stress concentrations, thereby minimizing perforation risk. ^11^ The favorable postoperative healing and clinical outcomes observed in the present study are consistent with previous reports by Ceruso et al., who demonstrated satisfactory clinical and radiographic outcomes following minimally invasive transcrestal sinus augmentation with simultaneous implant placement. ^12^ Furthermore, the ability to achieve predictable bone graft placement and simultaneous implant insertion with minimal bone manipulation supports improved surgical precision and patient tolerance. Collectively, these characteristics suggest that CAS-KIT assisted sinus elevation represents a logical refinement of existing transcrestal methodologies and may account for the favorable clinical and patient-reported outcomes observed, even in the absence of technique-specific comparative trials.

The uniform post-intervention scores with zero variability observed in this study indicate a high level of procedural reliability and reproducibility of the crestal approach technique. This consistency suggests predictable membrane elevation and graft stabilization with minimal technique-related variability. Although the use of specialized crestal systems involves an initial equipment cost and learning curve, these factors are balanced by improved patient comfort, reduced morbidity, shorter operative time, and fewer postoperative complications. Careful patient selection remains essential, with favorable outcomes primarily seen in cases with adequate residual bone height, favorable sinus anatomy, and absence of sinus pathology. When appropriately indicated, the crestal approach can be effectively integrated into treatment planning for posterior maxillary rehabilitation, facilitating minimally invasive sinus augmentation with simultaneous implant placement and enhanced patient-centered outcomes. The findings of the present study demonstrated favorable clinical healing and improvement in patient-reported oral health–related quality-of-life outcomes following CAS KIT–assisted indirect sinus augmentation and implant rehabilitation.

The present study has several limitations that warrant consideration. Its longitudinal observational design, with a single post-treatment assessment and absence of a control or comparison group, restricts causal inference and direct comparison with alternative sinus augmentation techniques. Although the sample size provided adequate statistical power, the single-center setting limits the generalizability of the findings. Additionally, the lack of long-term follow-up beyond the immediate post-prosthetic period precludes evaluation of the durability of patient-reported benefits. The reliance on subjective outcome measures without objective functional assessments, such as masticatory performance or nutritional evaluation, further limits comprehensive outcome interpretation, while retrospective baseline OHIP-14 assessment may be subject to recall bias. Also the present study is its single-center design with a relatively limited sample size, which may restrict the generalizability of the findings. Further multicenter studies with larger and more diverse populations are necessary to validate the present results. Future research should focus on well-designed comparative studies evaluating CAS KIT against conventional transcrestal and lateral window techniques, with extended follow-up. Incorporation of radiographic parameters, implant stability measures, economic evaluations, multicenter validation, and objective functional outcomes would strengthen the evidence base and better define the clinical value of minimally invasive sinus augmentation techniques.

## Conclusion

Within the limitations of this longitudinal observational study, CAS KIT–assisted indirect maxillary sinus augmentation followed by implant-supported rehabilitation resulted in significant improvement in oral health–related quality of life and patient-reported outcomes. The rapid postoperative recovery, minimal functional impairment, and uniform improvement in OHIP-14 scores reflect high patient satisfaction and favorable psychosocial impact associated with this minimally invasive approach. These findings suggest that the enhanced surgical control and reduced morbidity of CAS-KIT assisted sinus augmentation translate into meaningful patient-centered benefits. Incorporation of patient-reported outcome measures alongside clinical and radiographic parameters is essential for comprehensive evaluation of treatment success in posterior maxillary implant rehabilitation. Further comparative studies are warranted to validate these findings and assess long-term outcomes.

## Data Availability

RAGHER, M., SHETTY, S. K., SHETTY, R., DANDEKERI, S., M, S., & ABDUL AZIZ, N. (2026). Oral Health–Related Quality of Life and Patient-Reported Outcomes After Implant Rehabilitation Using CAS Kit–Assisted Indirect Maxillary Sinus Augmentation: A Longitudinal Observational Study [Data set]. Zenodo. https://doi.org/10.5281/zenodo.19274689 ^13^ Data are available under the terms of the Creative Commons Attribution 4.0 International license (CC-BY 4.0).
