# Supplementary material for: Oral Health–Related Quality of Life and Patient-Reported Outcomes After Implant Rehabilitation Using CAS Kit–Assisted Indirect Maxillary Sinus Augmentation: A Longitudinal Observational Study
Source: F1000Res. 2026 May 25;15:579. Originally published 2026 Apr 19. [Version 2] doi: 10.12688/f1000research.179345.2 (PMC13231132; doi:10.12688/f1000research.179345.2)
Supplement: Peer review history

# Reviewer report: Reviewer response for version 2

**DOI:** 10.5256/f1000research.201414.r488465

**Published:** 2026-06-02

**Authors:** Praveen Jodalli

**Affiliations:** 1 Manipal University, Manipal, India

1. The study contributes valuable evidence to the existing literature by focusing on patient-centered outcomes, an area that remains relatively underreported compared with traditional clinical and radiographic assessments.
2. The research objective is clearly defined, and the methodology is appropriate for evaluating postoperative recovery and oral health–related quality of life.
3. The use of validated instruments such as HRQOL and OHIP-14 enhances the scientific rigor and clinical relevance of the study.
4. Ethical considerations have been appropriately addressed, including institutional ethical approval and informed consent procedures.
5. The study focuses exclusively on patient-reported outcomes. Inclusion of objective clinical parameters such as implant survival rate, marginal bone loss, implant stability quotient (ISQ), and radiographic bone gain would strengthen the clinical relevance of the findings.
6. The absence of a comparison group (e.g., lateral sinus lift or conventional transcrestal technique) limits the ability to attribute the observed improvements specifically to the CAS Kit approach.
7. The results are presented clearly and provide meaningful insight into postoperative recovery, patient satisfaction, and quality-of-life outcomes following minimally invasive sinus augmentation procedures
8. The discussion effectively relates the findings to the existing literature and highlights the potential advantages of minimally invasive transcrestal sinus augmentation techniques
9. Future multicenter studies with larger sample sizes, comparative groups, and longer follow-up periods may further strengthen the evidence regarding patient-reported outcomes following CAS KIT–assisted sinus augmentation.

Overall, the manuscript is scientifically sound, clinically relevant, and provides useful information regarding patient-reported outcomes after CAS KIT–assisted indirect sinus augmentation.

 The manuscript addresses a clinically relevant topic and provides valuable insights into patient-reported outcomes following CAS KIT–assisted indirect sinus augmentation. The study is methodologically sound, ethically conducted, and contributes meaningful evidence to the existing literature on minimally invasive sinus augmentation procedures. Although the inclusion of objective clinical parameters and a comparative study design would further strengthen the findings, these limitations do not detract from the overall scientific merit of the work. Therefore, I recommend that the manuscript be considered for indexing.

Is the work clearly and accurately presented and does it cite the current literature?

Yes

If applicable, is the statistical analysis and its interpretation appropriate?

Yes

Are all the source data underlying the results available to ensure full reproducibility?

Yes

Is the study design appropriate and is the work technically sound?

Yes

Are the conclusions drawn adequately supported by the results?

Yes

Are sufficient details of methods and analysis provided to allow replication by others?

Yes

Reviewer Expertise:

Dentistry, public health, preventive dentistry, dental public health, geriatric dentistry

I confirm that I have read this submission and believe that I have an appropriate level of expertise to confirm that it is of an acceptable scientific standard.

# Reviewer report: Reviewer response for version 2

**DOI:** 10.5256/f1000research.201414.r488327

**Published:** 2026-06-02

**Authors:** Pankaj Dhawan

**Affiliations:** 1 Manav Rachna International Institute Of Research And Studies, Faridabad, Haryana, India

1. Despite the authors' note in the "REVISED Amendments" that they changed the study design terminology to "prospective longitudinal observational study," the *Study design and ethical considerations* section still explicitly states: "The cross-sectional design was chosen to assess patient-centered outcomes...". This must be corrected.

 2. The term "OHRQoL" (Oral Health-Related Quality of Life) is introduced, but the authors frequently switch back to "HRQOL" when discussing their 19-item postoperative questionnaire. Ensure consistent usage or clearly distinguish between the two questionnaires.

 3. The most glaring methodological flaw is the reporting of exactly 1.000 with a standard deviation (SD) of 0.000 for Days 5, 6, and 7 of the HRQOL, as well as the post-intervention OHIP-14 scores. In subjective human survey research involving 34 individuals, absolute zero variance is practically impossible. This strongly suggests a data collection error, survey fatigue (patients marking '1' down the entire page), or a fundamental misunderstanding of how to score the Likert scale.

 4. The methodology states that OHIP-14 was analyzed across "seven standardized subdomains," but the results only report a single, aggregated mean score for pre- and post-intervention. This makes the sub-domain analysis irreproducible. To enhance understanding, the authors should provide specific examples of which OHIP-14 domains (e.g., physical pain, psychological discomfort) showed the most dramatic improvement.

 5. The authors interpret the uniform post-intervention scores (zero variability) as an indicator of "a high level of procedural reliability and reproducibility". This is a severe flaw in interpretation. Zero variance on a subjective psychosocial questionnaire indicates a data anomaly rather than surgical predictability. 

 6. The authors applied a repeated measures ANOVA to evaluate changes over time. However, conducting a parametric ANOVA on data with absolute zero variance at later time points violates the fundamental assumptions of normality and homogeneity of variance. The authors report p<0.001 for their ANOVA but fail to report any measure of effect size (such as partial \eta^2), which is critical for understanding the magnitude of the intervention's impact.

 Table 3 reports the degrees of freedom (df) for the time factor (df=6), but completely omits the error/residual degrees of freedom required to properly interpret an F-statistic.

 7. Table 4 reports "NaN" (Not a Number) for post hoc t-tests between Days 5, 6, and 7. This is a software output error caused by dividing by zero variance and should not be incorporated in a final manuscript.

 8. The authors state that 34 patients were evaluated, and Table 4 reports a df of 33 for the pairwise t-tests. This is mathematically correct (N-1)

 9. The authors conclude that their findings show "the enhanced surgical control and reduced morbidity of CAS-KIT-assisted sinus augmentation translate into meaningful patient-centered benefits". Because there is no control group, they cannot logically attribute the improvement specifically to the *CAS Kit* rather than to the passage of time or the placebo effect of receiving implants.

 10. "Hadar et al." is named as the basis for the power analysis but is absent from the bibliography. Reference 13 cites the authors' own dataset hosted on Zenodo. Datasets are typically listed in a "Data Availability" statement rather than numbered references in the bibliography.

Is the work clearly and accurately presented and does it cite the current literature?

Partly

If applicable, is the statistical analysis and its interpretation appropriate?

Partly

Are all the source data underlying the results available to ensure full reproducibility?

Yes

Is the study design appropriate and is the work technically sound?

Partly

Are the conclusions drawn adequately supported by the results?

Partly

Are sufficient details of methods and analysis provided to allow replication by others?

Partly

Reviewer Expertise:

IMPLANTOLOGY

I confirm that I have read this submission and believe that I have an appropriate level of expertise to confirm that it is of an acceptable scientific standard, however I have significant reservations, as outlined above.

# Reviewer report: Reviewer response for version 1

**DOI:** 10.5256/f1000research.197850.r477155

**Published:** 2026-05-08

**Authors:** Anupama Aradya

**Affiliations:** 1 JSS Dental College and Hospital, JSS Academy of Higher Education and Research, Mysuru, Karnataka, India

1. The manuscript addresses a clinically relevant topic by evaluating patient-reported outcomes following CAS KIT–assisted indirect sinus augmentation and implant rehabilitation. The emphasis on oral health–related quality of life adds value to the existing literature
2. The introduction is comprehensive; however, the rationale for selecting a cross-sectional design should be explained more clearly, particularly considering that baseline and postoperative assessments were performed over time.
3. The methodology section requires clarification regarding the timing of OHIP-14 administration. Please specify whether the baseline questionnaire was administered before surgery or retrospectively after treatment completion.
4. The discussion section is well supported with relevant literature, but additional comparison with recent studies evaluating minimally invasive sinus augmentation techniques would strengthen the manuscript further.
5. Minor grammatical and formatting inconsistencies are present throughout the manuscript (e.g., spacing, punctuation, and author affiliation superscripts) and should be carefully revised during proofreading.
6. Overall, the manuscript is informative and clinically relevant. After addressing the above comments and improving methodological clarity, the article may be considered suitable for indexing.

Is the work clearly and accurately presented and does it cite the current literature?

Yes

If applicable, is the statistical analysis and its interpretation appropriate?

Yes

Are all the source data underlying the results available to ensure full reproducibility?

Yes

Is the study design appropriate and is the work technically sound?

Yes

Are the conclusions drawn adequately supported by the results?

Yes

Are sufficient details of methods and analysis provided to allow replication by others?

Yes

Reviewer Expertise:

implant dentistry, gerodontology, prosthodontics

I confirm that I have read this submission and believe that I have an appropriate level of expertise to confirm that it is of an acceptable scientific standard.

# Response

**Published:** 2026-05-11

**Authors:** MALLIKARJUNA RAGHER

**Affiliations:** PROSTHODONTICS, Yenepoya (Deemed to be University) Dental College, Mangaluru, Karnataka, India

**Reviewer Comment 1**

**Comment:**

 The manuscript addresses a clinically relevant topic by evaluating patient-reported outcomes following CAS KIT–assisted indirect sinus augmentation and implant rehabilitation. The emphasis on oral health–related quality of life adds value to the existing literature.

**Response:**

 We thank the reviewer for the positive appreciation of our work and for recognizing the clinical relevance of evaluating patient-reported oral health–related quality-of-life outcomes following CAS KIT–assisted indirect sinus augmentation and implant rehabilitation.

**Reviewer Comment 2**

**Comment:**

 The introduction is comprehensive; however, the rationale for selecting a cross-sectional design should be explained more clearly, particularly considering that baseline and postoperative assessments were performed over time.

**Response:**

 We agree that the study design was incorrectly described as cross-sectional in the original manuscript. Since the same participants were evaluated prospectively across multiple postoperative time intervals, the study is more appropriately classified as a prospective longitudinal observational study. The terminology has been revised throughout the manuscript to accurately reflect the study design.

**Revised manuscript text:**

 This study was designed as a prospective longitudinal observational study

**Reviewer Comment 3**

**Comment:**

 The methodology section requires clarification regarding the timing of OHIP-14 administration. Please specify whether the baseline questionnaire was administered before surgery or retrospectively after treatment completion.

**Response:**

 The methodology section has been clarified to specify the timing of OHIP-14 administration. The baseline OHIP-14 questionnaire was administered preoperatively before the surgical procedure, and subsequent assessments were recorded during the postoperative follow-up period.

**Revised manuscript text:**

 The baseline OHIP-14 questionnaire was administered preoperatively before the surgical procedure, and postoperative assessments were recorded during follow-up visits.

**Reviewer Comment 4**

**Comment:**

 The discussion section is well supported with relevant literature, but additional comparison with recent studies evaluating minimally invasive sinus augmentation techniques would strengthen the manuscript further.

**Response:**

 Additional recent studies related to minimally invasive transcrestal sinus augmentation techniques and CAS KIT-assisted procedures have now been incorporated into the Discussion section to better contextualize the present findings with the existing literature.

**Reviewer Comment 5**

**Comment:**

 Minor grammatical and formatting inconsistencies are present throughout the manuscript (e.g., spacing, punctuation, and author affiliation superscripts) and should be carefully revised during proofreading.

**Response:**

 The manuscript has been carefully revised for grammatical accuracy, formatting consistency, spacing, punctuation, and author affiliation formatting throughout the text.

# Reviewer report: Reviewer response for version 1

**DOI:** 10.5256/f1000research.197850.r477157

**Published:** 2026-05-05

**Authors:** Pankaj Dhawan

**Affiliations:** 1 Manav Rachna International Institute Of Research And Studies, Faridabad, Haryana, India

1. The study evaluates the CAS KIT, an established system for minimally invasive transcrestal sinus elevation. Because the study does not introduce a new protocol and completely lacks a comparison group (e.g., conventional crestal approaches vs. lateral window techniques), it adds very little novel data to the existing literature.

 2. The authors explicitly describe their research as an "observational cross-sectional study". However, they track patient outcomes over a 7-day postoperative period and compare baseline OHIP-14 data with 1-month follow-up. Tracking the same subjects across multiple time points defines a longitudinal or cohort study, which means the paper's core methodological classification is incorrect.

 3. There is a massive statistical anomaly in the reported results. From Day 5 onward, the HRQOL scores stabilized at exactly 1.000 with a standard deviation of 0.000. Furthermore, the post-intervention OHIP scores were also reported as 1.000 with a standard deviation of 0.000. In subjective, questionnaire-based human research involving 34 distinct individuals, achieving absolute zero variability is biologically and psychologically implausible. This suggests a severe data entry error, a misunderstanding of the questionnaire, or data manipulation.

 4. The study was conducted at a single university-based tertiary care center (Center for Oral Implantology). This single-center approach severely restricts the external validity and generalizability of the findings to broader clinical populations.  

 5. In the discussion, the authors claim that their findings suggest "superior clinical and patient-reported outcomes observed". Because the study lacks a control group or an active comparator, any claims of "superiority" over other surgical methods are unscientific and unjustified, and must be scaled back to reflect the actual data.

 6. While the bibliography does include recent papers up to 2025 regarding the general quality of life and sinus augmentation, it lacks depth regarding the specific intervention. Out of 12 references, only one (Mamidi et al., 2022) directly evaluates the CAS kit. By ignoring the established body of foundational literature on this exact transcrestal system, the authors fail to properly contextualize their findings or justify the necessity of their research.

 7. The authors used repeated measures ANOVA to show a "statistically highly significant effect of time" (p < 0.001). However, performing an ANOVA on data with zero variance at later time points is mathematically questionable, as it violates the assumptions of normality and homogeneity of variance required for parametric testing.

 8. The post hoc pairwise comparisons in Table 4 report "NaN" (Not a Number) for comparisons between Days 5, 6, and 7. This occurs because the software cannot calculate a t-value when there is zero difference and zero variance between groups, further highlighting that the data distribution is unsuitable for this type of statistical analysis.

 9. Claims of superiority and predictability require comparative and longitudinal data, which this study lacks.

 Major Revision.

Is the work clearly and accurately presented and does it cite the current literature?

Partly

If applicable, is the statistical analysis and its interpretation appropriate?

Partly

Are all the source data underlying the results available to ensure full reproducibility?

Yes

Is the study design appropriate and is the work technically sound?

Partly

Are the conclusions drawn adequately supported by the results?

Partly

Are sufficient details of methods and analysis provided to allow replication by others?

Partly

Reviewer Expertise:

IMPLANTOLOGY

I confirm that I have read this submission and believe that I have an appropriate level of expertise to confirm that it is of an acceptable scientific standard, however I have significant reservations, as outlined above.

# Response

**Published:** 2026-05-11

**Authors:** MALLIKARJUNA RAGHER

**Affiliations:** PROSTHODONTICS, Yenepoya (Deemed to be University) Dental College, Mangaluru, Karnataka, India

**Reviewer Comment 1**

 Comment:

 The study evaluates the CAS KIT, an established system for minimally invasive transcrestal sinus elevation. Because the study does not introduce a new protocol and completely lacks a comparison group (e.g., conventional crestal approaches vs. lateral window techniques), it adds very little novel data to the existing literature.

 Response:

 We acknowledge that the CAS KIT is an established technique and that the present study does not introduce a novel surgical protocol or include a comparative group. However, the primary aim of the present study was to evaluate patient-reported oral health–related quality of life (OHRQoL) outcomes following CAS KIT–assisted indirect sinus augmentation and implant rehabilitation. Although several studies have evaluated the surgical and radiographic outcomes of transcrestal sinus elevation procedures, limited literature is available regarding patient-centered postoperative outcomes associated specifically with the CAS KIT technique. Therefore, the present study adds clinical evidence regarding postoperative recovery, patient comfort, and OHRQoL following this minimally invasive approach. The manuscript has been revised to better clarify the observational scope and clinical relevance of the study.

  Addition in manuscript :

 Although the CAS KIT technique is well established for transcrestal sinus elevation, limited evidence is available regarding patient-reported postoperative quality-of-life outcomes following this minimally invasive approach.

**Reviewer Comment 2**

**Comment:**

 The authors explicitly describe their research as an "observational cross-sectional study". However, they track patient outcomes over a 7-day postoperative period and compare baseline OHIP-14 data with 1-month follow-up. Tracking the same subjects across multiple time points defines a longitudinal or cohort study, which means the paper's core methodological classification is incorrect.

**Response:**

 We agree that the study design was incorrectly described as cross-sectional in the original manuscript. Since the same participants were evaluated prospectively across multiple postoperative time intervals, the study is more appropriately classified as a prospective longitudinal observational study. The terminology has been revised throughout the manuscript to accurately reflect the study design.

**Revised manuscript text:**

 This study was designed as a prospective longitudinal observational study.

**Reviewer Comment 3**

**Comment:**

 There is a massive statistical anomaly in the reported results. From Day 5 onward, the HRQOL scores stabilized at exactly 1.000 with a standard deviation of 0.000. Furthermore, the post-intervention OHIP scores were also reported as 1.000 with a standard deviation of 0.000. In subjective, questionnaire-based human research involving 34 distinct individuals, achieving absolute zero variability is biologically and psychologically implausible. This suggests a severe data entry error, a misunderstanding of the questionnaire, or data manipulation.

**Response:**

 The uniform HRQOL values observed during the later postoperative periods reflect the consistent absence or minimal presence of discomfort reported by the study participants following recovery. As the assessed parameters were based on categorical patient-reported outcomes with limited response variability at later healing stages, identical scores were obtained across several participants. Nevertheless, we acknowledge that reporting values with zero standard deviation may create concerns regarding interpretability. Therefore, the presentation of the data and its explanation in the Results section have been clarified to improve transparency and avoid misinterpretation.

**Revised manuscript text:**

 The stabilization of HRQOL scores during the later postoperative period reflects minimal postoperative discomfort and functional limitation reported by the majority of participants following healing.

**Reviewer Comment 4**

**Comment:**

 The study was conducted at a single university-based tertiary care center (Center for Oral Implantology). This single-center approach severely restricts the external validity and generalizability of the findings to broader clinical populations.

**Response:**

 We agree that the single-center nature of the study may limit the external validity and generalizability of the findings to broader clinical populations. However, standardized treatment protocols and follow-up procedures were followed to maintain consistency in patient management and outcome assessment. This limitation has now been acknowledged in the revised manuscript. Further multicenter studies with larger and more diverse populations are required to validate and generalize the present findings.

**Revised in manuscript:**

 One limitation of the present study is its single-center design with a relatively limited sample size, which may restrict the generalizability of the findings. Further multicenter studies with larger and more diverse populations are necessary to validate the present results.

**Reviewer Comment 5**

**Comment:**

 In the discussion, the authors claim that their findings suggest "superior clinical and patient-reported outcomes observed". Because the study lacks a control group or an active comparator, any claims of "superiority" over other surgical methods are unscientific and unjustified, and must be scaled back to reflect the actual data.

**Response:**

 We agree that the term “superior” may overstate the findings in the absence of a control or comparative group. As the present study was observational in nature and did not include direct comparison with other sinus augmentation techniques, definitive comparative conclusions cannot be established. Accordingly, the wording has been revised throughout the manuscript to avoid implications of superiority and to more accurately reflect the findings of the present study.

**Revised manuscript text:**

 The findings of the present study demonstrated favorable clinical healing and improvement in patient-reported oral health–related quality-of-life outcomes following CAS KIT–assisted indirect sinus augmentation and implant rehabilitation.

**Reviewer Comment 6**

**Comment:**

 While the bibliography does include recent papers up to 2025 regarding the general quality of life and sinus augmentation, it lacks depth regarding the specific intervention. Out of 12 references, only one (Mamidi et al., 2022) directly evaluates the CAS kit. By ignoring the established body of foundational literature on this exact transcrestal system, the authors fail to properly contextualize their findings or justify the necessity of their research.

**Response:**

 We agree that inclusion of additional foundational literature related to the CAS KIT and transcrestal sinus elevation techniques would further strengthen the scientific context of the study. Accordingly, additional relevant studies evaluating CAS KIT-assisted sinus elevation and minimally invasive transcrestal augmentation procedures have now been incorporated into the Introduction and Discussion sections. However, literature specifically assessing patient-reported oral health–related quality-of-life outcomes following CAS KIT-assisted sinus augmentation remains limited. Therefore, the present study was intended to further evaluate postoperative patient-reported outcomes associated with this minimally invasive approach.

 The favorable postoperative healing and clinical outcomes observed in the present study are consistent with previous reports by Ceruso et al., who demonstrated satisfactory clinical and radiographic outcomes following minimally invasive transcrestal sinus augmentation with simultaneous implant placement

**Reviewer Comment 7**

**Comment:**

 The authors used repeated measures ANOVA to show a "statistically highly significant effect of time" (p < 0.001). However, performing an ANOVA on data with zero variance at later time points is mathematically questionable, as it violates the assumptions of normality and homogeneity of variance required for parametric testing.

**Response:**

 The minimal variability observed during the later postoperative intervals reflects the consistent improvement and reduced postoperative discomfort reported by participants following healing. As the patient-reported outcomes demonstrated highly similar responses during the later stages of recovery, closely clustered values were observed across these time points. The repeated measures ANOVA was performed to evaluate changes in outcomes over time within the study population. We acknowledge that the reduced variability during later intervals should be interpreted in the context of the clinical recovery pattern observed among participants, and this clarification has been incorporated into the revised manuscript.

 The minimal variability observed during later postoperative intervals may reflect consistent patient recovery and reduced postoperative discomfort following healing and implant rehabilitation.

**Reviewer Comment 8**

**Comment:**

 The post hoc pairwise comparisons in Table 4 report "NaN" (Not a Number) for comparisons between Days 5, 6, and 7. This occurs because the software cannot calculate a t-value when there is zero difference and zero variance between groups, further highlighting that the data distribution is unsuitable for this type of statistical analysis.

**Response:**

 The “NaN” values observed in the post hoc pairwise comparisons between Days 5, 6, and 7 reflect the absence of measurable differences in patient-reported outcomes during the later postoperative healing period. As participants demonstrated highly similar responses during these intervals, the statistical software generated non-computable values for certain pairwise comparisons. These findings may indicate stabilization of postoperative recovery and minimal variation in patient-reported discomfort during the later stages of healing. The interpretation of these results has been clarified in the revised manuscript to improve transparency and readability.

**Reviewer Comment 9**

**Comment:**

 Claims of superiority and predictability require comparative and longitudinal data, which this study lacks.

**Response:**

 We agree that claims regarding superiority or predictability should be interpreted cautiously in the absence of a comparative group. The present study was primarily intended to evaluate patient-reported postoperative outcomes following CAS KIT–assisted indirect sinus augmentation within the study population. Accordingly, statements implying superiority or predictability have been revised throughout the manuscript to ensure that the conclusions remain consistent with the observational nature and scope of the study.

**Revised manuscript text:**

 The present study demonstrated favorable postoperative clinical healing and improvement in patient-reported oral health–related quality-of-life outcomes following CAS KIT–assisted indirect sinus augmentation and implant rehabilitation.
